# Propagation and attenuation characteristics of shock waves in a gas–coal dust explosion in a diagonal pipeline network

**DOI:** 10.1038/s41598-022-17199-8

**Published:** 2022-07-27

**Authors:** Jinzhang Jia, Xiuyuan Tian

**Affiliations:** 1 College of Safety Science and Engineering, Liaoning Technical University, Fuxin, 123000 Liaoning China; 2 Key Laboratory of Mine Thermal Power Disaster and Prevention, Ministry of Education, Fuxin, 123000 Liaoning China

**Keywords:** Natural hazards, Engineering

## Abstract

In order to obtain the propagation and attenuation characteristics of a shock wave gas–coal dust explosion, a diagonal pipeline network was built to carry out a gas–coal dust explosion experiment. Methane with a volume fraction of 9.5% was mixed with 20 g of coal dust with a particle size of 50 μm. The shock wave attenuation characteristics in the diagonal pipeline network were characterized by the overpressure attenuation coefficient. The results showed that when the shock wave propagated in the diagonal pipeline network, it was offset and superposed multiple times. The overpressure attenuation in diagonal pipes was greater than that in the other pipeline branches. Secondary explosions occurred at all monitoring points. The propagation characteristics of shock waves is less affected by the pipeline network structure when it is close to the outlet of pipeline network. Nonlinear multivariate regression analysis was carried out to analyze the relationship between the overpressure attenuation coefficient and two adjacent monitoring points, the pressure before and after attenuation, and the pipeline angle. The functional relationship was in good agreement with the experiment data.

## Introduction

With the increase of the coal mining depth, there are increased gas emissions. During the coal mining process, a large amount of coal dust is generated and suspended in the roadway^1,2^. Compared with a gas explosion^3,4^, a gas–coal dust explosion is more complex, and this type of explosion can produce greater explosion overpressure, resulting in multiple explosions and secondary disasters and endangering the lives of underground coal workers^5,6^.

Many studies have been carried out in recent years, both experimentally and numerically, on gas–coal dust explosion characteristics, as well as the influencing factors such as the coal dust and gas concentrations, baffle settings, and pipeline types. It was found that the addition of low-concentration coal dust increased the pressure and flame propagation speed of the explosions, and the volatile content and gas content in the coal dust played a large role in the explosions^7–10^. Moreover, there were interactions between the baffles and the gas–coal dust explosion. The explosion overpressure was significantly affected by the size of the obstacle and the blockage condition of the pipeline^11–13^. In particular, the effect of the pipeline type has received extensive attention in recent years^14,15^. For example, Arndt et al.^16^ reported that a 90-degree bend had the ability to enhance flame speeds and overpressures and shorten the run-up distance to DDT (Deflagration-to-Detonation transition) to a varying degree for several gases. Niu et al.^17^ studied the explosive overpressure evolution and flame propagation characteristics in transversal pipeline networks. The results showed that in the parallel branches, the overpressure peak and flame propagation velocity gradually decreased as the distance from the ignition point increased. Qi et al.^18^ measured the maximum explosion pressure, explosion index, and lower explosion limit of coal dust and gas mixtures in a standard 20 L spherical explosion system. It is concluded that adding a certain mass concentration of gas to the coal dust cloud can greatly increase the maximum explosion pressure and explosion index, and significantly reduce the minimum explosion concentration of coal dust powder. Li et al.^19^ studied the effects of coal dust powder injection pressure and ignition delay time on the explosion parameters of methane coal mixture in a confined space. The results indicated that the particle dispersion and stabilization time are significantly different under different injection pressures. These results are helpful to prevent methane coal particle explosion. Zhu et al.^20^ studied the explosion flow field in five straight pipes with different diameters and one curved pipeline using a numerical simulation. The results showed that in both the straight and bending pipes, the pressure wave and the velocity wave were accelerated by the rise of the reaction rate.

In summary, most of the previous studies on gas–coal dust explosion were carried out on simple bifurcated pipelines, long straight pipelines, or spherical explosion tanks^18,21–25^. These pipeline networks are not similar to an underground roadway. Thus, the conclusions of these studies are not applicable to an underground coal mine explosion. A coal mine roadway system is a complex network, and the shock waves generated by a gas–coal dust explosion will interact with and impact each other in the roadway network, resulting in offset and superposition effects. The interaction will aggravate the complexity of the shock wave propagation. Thus, it is necessary to study the influence of a pipeline structure on shock wave propagation. Therefore, in this study, parallel pipelines, bifurcated pipelines, and angle-connected pipelines were combined to study the propagation and attenuation characteristics of shock waves in a gas–coal dust explosion in order to provide theoretical references for the prevention and control of gas–coal dust explosions as well as the formulation of post-disaster emergency rescue plans.

## Experimental system and physicochemical properties of coal samples

### Experimental system

**Figure 1:**
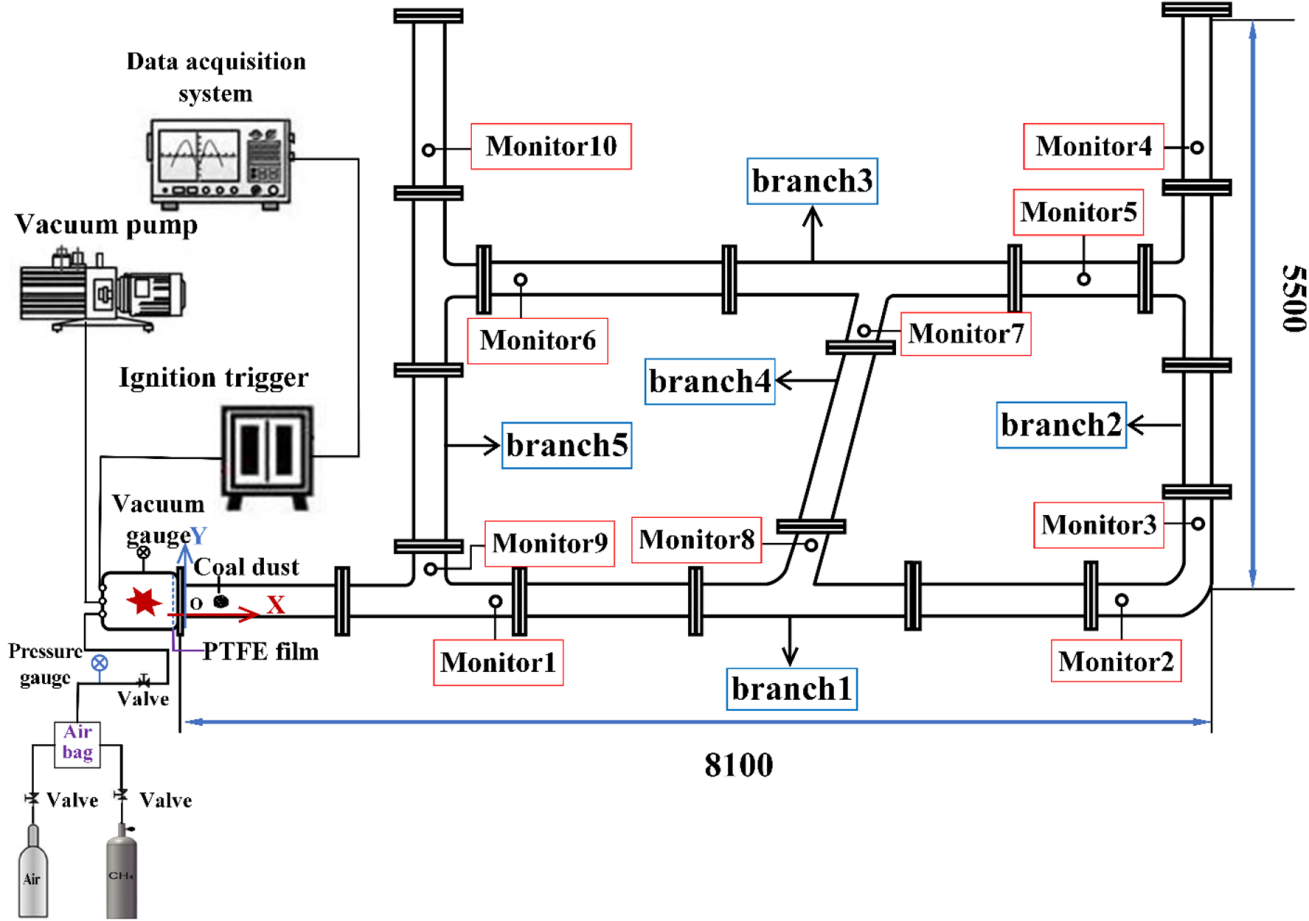
Experimental system.

As shown in Fig. 1, the size of the experimental pipe network was 8100 mm × 5500 mm. The experimental system was composed of a pipeline network and an explosion chamber. The volume of explosion chamber is 0.5 m^3^, the inner diameter of each pipeline is 500 mm, and the pressure resistance is more than 20 MPa^26^. The pipes in the pipeline network were connected with screws, the outlets of the pipeline networks were closed with flanges, and threaded holes were used to install sensors on the pipes. Sealing gaskets were installed at each pipe joint, to ensure that the equipment was properly sealed. Polytetrafluoroethylene film was also used to separate the explosion chamber from the pipes.

A TST6300 data acquisition system was used to collect the data in real time, which mainly consisted of a CYG1721 high precision pressure sensor. The response times of the pressure sensors were 1 ms, the accuracy of the data acquisition device was 0.2% FS, and the continuous acquisition frequency was 10 kHz/CH (channel). The ignition system mainly included a DX-GDH high-energy igniter, high-energy sparkplugs, high-voltage-resistant and high-temperature-resistant cables, power supply cables, and external trigger devices. The ignition control box was connected with an external trigger wire. The sparkplugs were placed at the front end of the explosion chamber, the ignition voltage was about 2200 V, and the one-time energy storage was 30 J^3^.

After the explosion chamber was vacuumed, the experimental gas with a volume fraction of 9.5% was passed through the chamber, the coal dust samples used in the experiment were evenly laid in the pipeline, and atmospheric air was piped into the pipe. The ignition device is controlled by computer, and the explosion experiment is carried out after 5 s of ignition delay^3,27^.

A coordinate system was established by taking the right end of the gas explosion chamber as the origin, the horizontal direction as the *x*-axis, and the vertical direction as the *y*-axis. The coordinates of each monitoring point are shown in Table 1.

**Table 1:** Coordinates of each monitoring point.

Monitoring point	*T*_1_	*T*_2_	*T*_3_	*T*_4_	*T*_5_	*T*_6_	*T*_7_	*T*_8_	*T*_9_	*T*_*10*_
Coordinates (m)	2.5, 0.1	7.4, 0.1	8.0, 0.8	8.0, 4.3	7.1, 3.1	2.7, 3.1	5.4, 2.6	5.0, 0.7	2.0, 0.4	2.0, 4.3

### Physicochemical properties of coal samples

Some studies have shown that among the different types of coal dust, coking coal has the most violent combustions and explosion reactions. Therefore, the coal samples in this study were coking coal from the Sihe Coal Mine, China. The coal dust had a mass of 20 g, a particle size of 50 μm, and an explosion index of 31.56% (an index of over 15% is considered explosive)^27^. The industrial analysis of the coal samples showed that the moisture content, ash content, and volatile content were 1.56%, 12.06%, and 30.06%, respectively.

**Figure 2:**
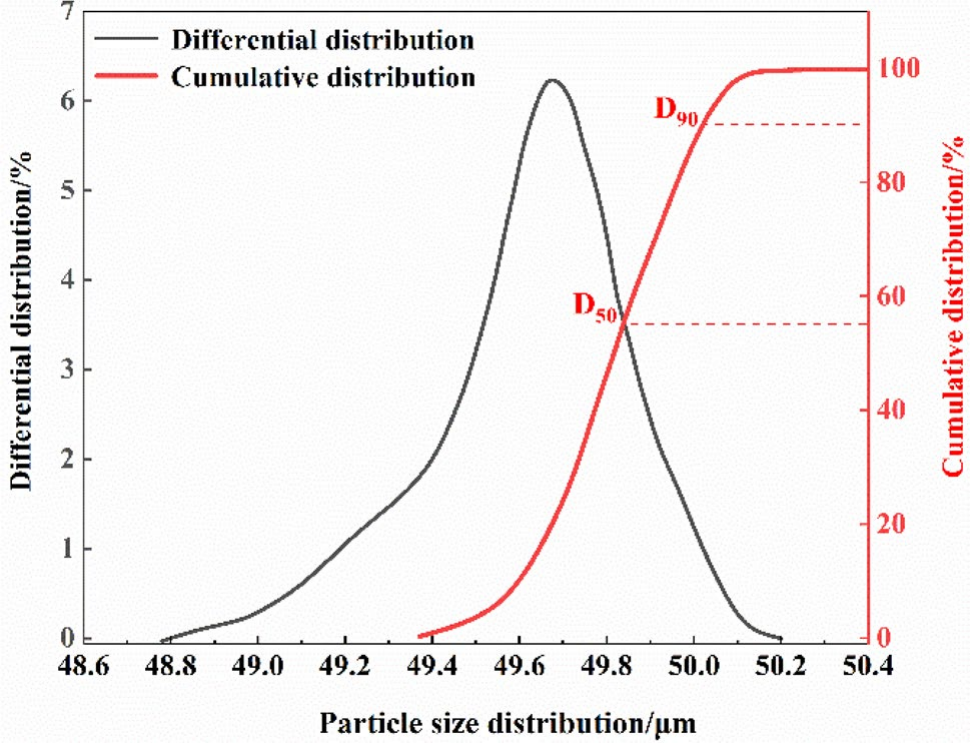
Particle size distribution.

**Figure 3:**
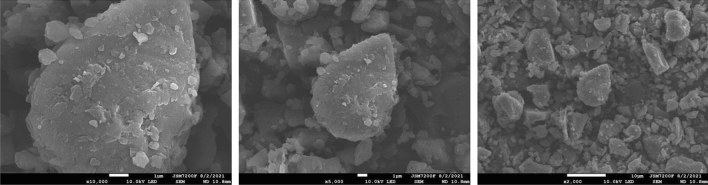
Scanning electron microscope of coal dust sample.

The analysis results of coal dust particle size distribution in Fig. 2 indicate that the particle size distribution of coal dust samples with more than 90% of coal dust samples used in the experiment can meet the experimental requirements. Figure 3 shows the scanning electron microscope results of the coal dust samples used in the experiment. The shape and size distribution of coal dust particles are uneven, the flake structure of coal dust samples is clear and the pore structure is not damaged. Therefore, the conclusion that the coal dust sample we selected is suitable can be obtained.

## Results analysis and discussion

### Shock wave evolution

**Figure 4:**
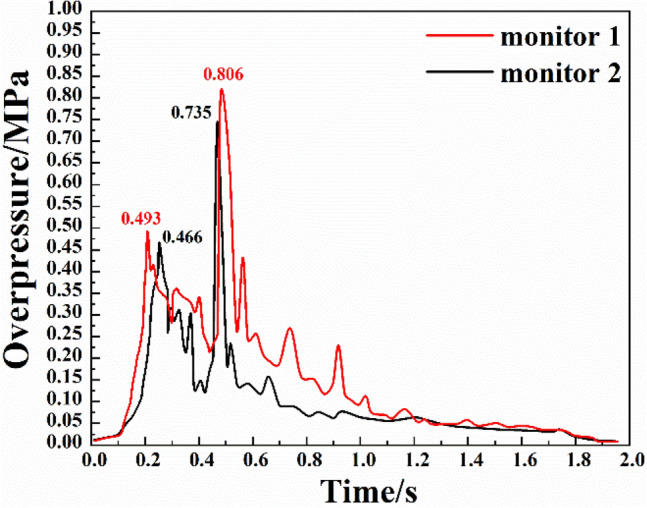
Overpressure change of bifurcation 1.

The variations of the overpressure at two monitoring points in branch pipeline 1 are shown in Fig. 4. It can be seen from the figure that:

1. Due to the complex pipeline network, the overpressure wave in branch pipeline 1 experienced multiple superpositions and attenuations during the propagation process. The first overpressure peaks at the two monitoring points both appeared in the initial stage of the reaction, followed by the second overpressure peaks, which were significantly larger than the first peaks. Thus, there was a secondary explosion in branch pipeline 1. The violent reactions mainly occurred in the early stage of the explosion, and in the later stage, there were slight fluctuations due to the reverse shock wave from other pipelines.
2. The evolution of the shock waves at monitoring point 1 could be divided into four stages. The first stage was the initial explosion period, in which the overpressure duration was short, and the pressure did not change significantly. The second stage was the sudden change period. Due to the detonation reaction of the gas dust and the coal dust, the shock wave propagated forward, resulting in an increase in the duration of the overpressure, and the overpressure rose significantly with the simultaneous actions of the compression wave and the flame wave. The first overpressure peak of 0.493 MPa was reached at 0.209 s. The third stage was the secondary explosion period, in which the overpressure first dropped and then fluctuated for a period of time. Then the coal dust cloud was decomposed under high-temperature and high-pressure conditions, producing a sufficient amount of combustible gas to resume the reaction. As a result, the overpressure increased again, and the secondary overpressure peak of 0.806 MPa appeared at 0.480 s. The last stage was the stable attenuation period. The overpressure fluctuated due to the small impact of shock waves from other pipelines and then entered a steady attenuation state.
3. The variation of overpressure at monitoring point 2 was similar to that at monitoring point 1. However, the two overpressure peaks at measuring point 2 were lower than those at monitoring point 1, which were 0.466 MPa and 0.735 MPa. Thus, the overpressure showed obvious attenuation after passing the bifurcated pipelines in the complex pipeline network. In the middle and late stages of the explosion, the overpressure at monitoring point 2 was smaller than that at monitoring point 1 and the fluctuation was lower. This was because the position of monitoring point 2 was relatively near the back; that is, it was less affected by shock waves in the other branch pipes compared to monitoring point 1. The distance between the monitoring point and the explosion cavity is closely related to the value of the peak value of the explosion overpressure at the monitoring point, but is less related to the rate of explosion overpressure rise. It can also be seen from the experimental results that the two overpressure peaks at monitoring point 1 are significantly higher than those at monitoring point 2.

**Figure 5:**
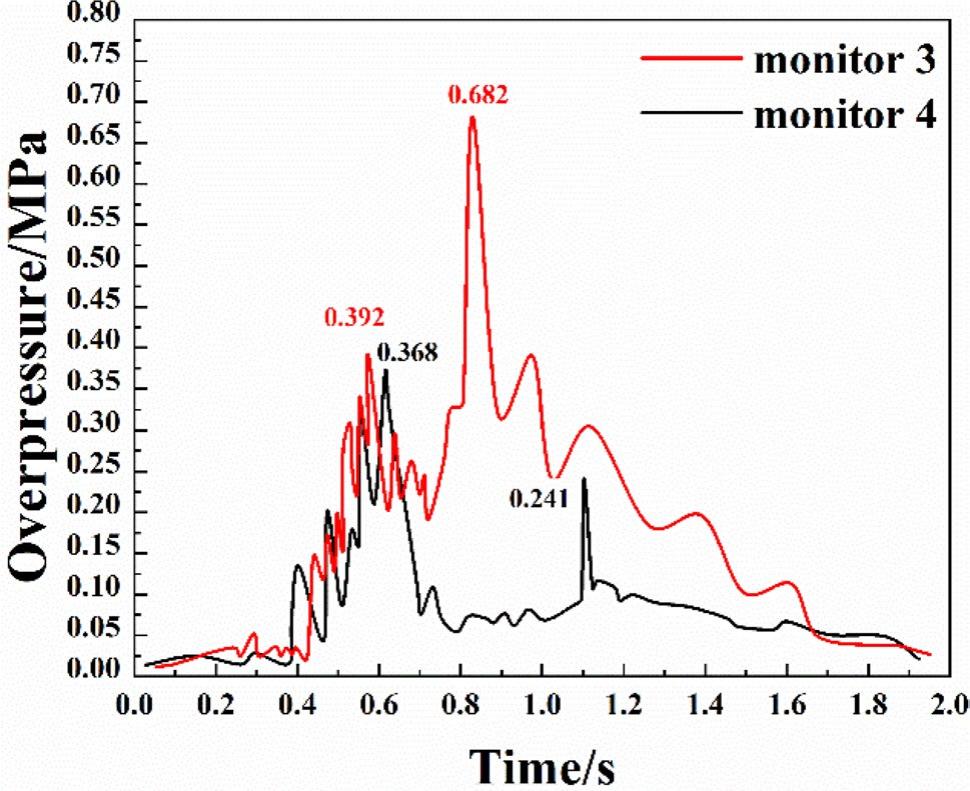
Overpressure change of bifurcation 2.

The variations of the overpressure at the two monitoring points in branch pipeline 2 are shown in Fig. 5. It can be seen from the figure that:

1. Compared with the monitoring points in branch pipeline 1, the superposition and attenuation of the overpressure in branch pipeline 2 increased slightly during propagation, and the number of overpressure peaks in the whole process increased. Moreover, the period between the first and second overpressure peaks was increased, and the durations of the peaks were longer. The strength was mainly concentrated in the middle stage of the explosion, which was mainly due to the position of the branch pipe.
2. The first stage of the evolution of the shock waves at monitoring point 3 was the initial explosion period in which there were small fluctuations of the overpressure that lasted for about 0.4 s. The fluctuation was mainly caused by multiple shock waves in other pipelines. The second stage was the sudden change period. With the combustion reaction of gas and coal dust, the overpressure continuously propagated forward and reached the first peak of 0.392 MPa at 0.573 s. The third stage was the secondary explosion period. The overpressure started to rise again and reached the second peak of 0.682 MPa at 0.829 s. The secondary explosion was mainly because some coal dust absorbed heat and reached an explosive concentration. The fourth stage was the stable attenuation period. As the reaction progressed, the overpressure continuously fluctuated and declined until the end of the reaction.
3. During the whole process, the overpressure at monitoring point 4 was similar to that at point 3, with some differences. Within the first 0.4 s, the overpressure fluctuation at point 4 was smaller, and then there was a sharp rise in the overpressure. The overpressure reached the first peak of 0.368 MPa at 0.615 s after several up-down cycles. Then, due to the secondary explosion, the overpressure first decreased and then increased, reaching the second peak of 0.241 MPa at 1.10 s. Due to the position of monitoring point 4 (i.e., close to the explosion vent and far from the explosion source), the overpressure was attenuated after passing through branch pipeline 2, and with the oxygen consumption, the second overpressure peak was lower than the first one and appeared at a later time.

**Figure 6:**
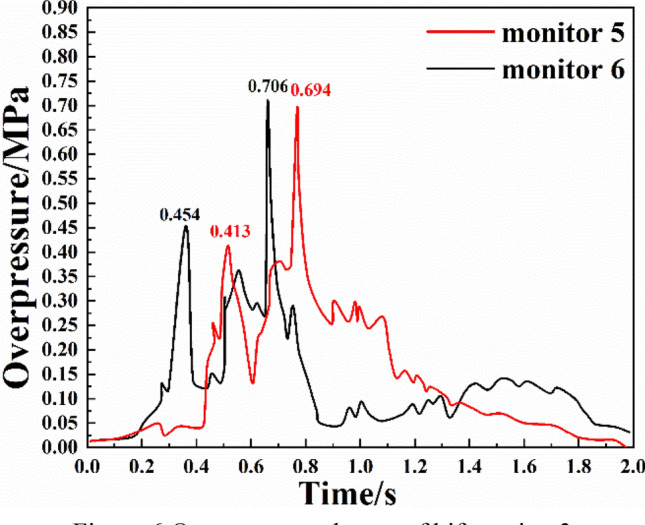
Overpressure change of bifurcation 3.

The variations of the overpressure at two monitoring points in branch pipeline 3 are shown in Fig. 6. It can be seen from the figure that:

1. The number of superposition and attenuation of shock waves in branch pipeline 3 was not significantly higher than that in branch pipeline 2, and the number of extreme overpressure values in the entire process was close to that for branch pipeline 2. However, the number of up-down cycles of the overpressure in branch pipeline 3 increased slightly. The time of an overpressure peak was similar at the two monitoring points. Since branch pipeline 3 was connected with more pipelines, the overpressure was more affected by the shock waves from the other pipelines.
2. The first stage of the shock wave evolution at monitoring point 5 was the initial explosion period, which was about 0.2 s. The second stage was the sudden change period in which the overpressure propagated forward and passed through two bends. The first overpressure peak of 0.413 MPa appeared at 0.517 s. The third stage was the secondary explosion period, and the overpressure rose rapidly. Due to the incomplete gas–coal dust reaction and the high temperature, a residual reaction was triggered. The second overpressure peak of 0.694 MPa was reached at 0.769 s. The fourth stage was the stable attenuation period, in which the shock waves gradually attenuated and the overpressure dropped rapidly.
3. The evolution of the shock waves at monitoring point 6 was similar to that at monitoring point 5. However, monitoring point 6 was less affected by shock waves from the other pipelines, and the overpressure reached the first peak value of 0.454 MPa at 0.362 s. Because point 6 was closer to the explosion source than point 5, the first peak was higher and earlier than the first peak at point 5. The secondary overpressure peak of 0.706 MPa was reached at 0.661 s. At 0.929 s, the overpressure dropped to the lowest value of 0.005 MPa due to the pressure relief at the explosion vent. After that, the overpressure fluctuated slightly due to the influence of the shock waves from the other pipelines, but the overall change was small. Based on the experimental results, it can be inferred that because there are many pipelines connected to the branch pipe 3 where the monitoring point 6 is located, and the monitoring point 6 is closer to the explosion cavity than the monitoring point 5. Therefore, in the process of explosion shock wave evolution, the fluctuation is more significant due to the influence of strong shock waves in other pipelines. Similar phenomena have also occurred at other monitoring points (such as monitoring point 3 and monitoring point 7), but they are not significantly reflected because the later fluctuations are too weak.

**Figure 7:**
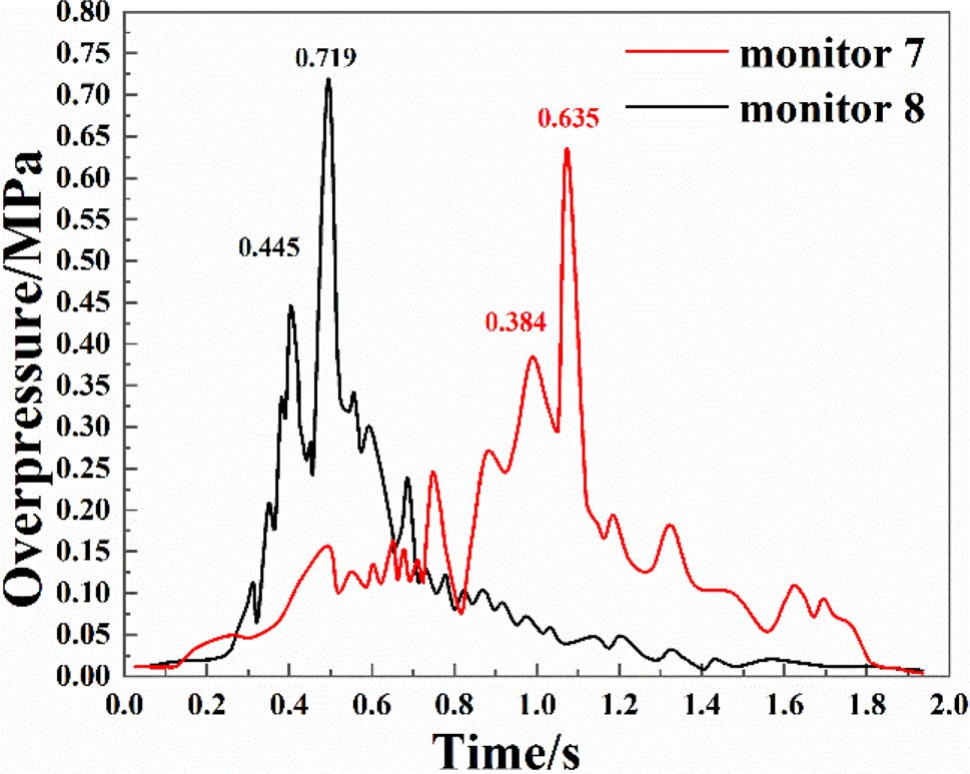
Overpressure change of bifurcation 4.

The variations of the overpressure at two monitoring points in branch pipeline 4 are shown in Fig. 7. It can be seen from the figure that:

1. The first overpressure peak at monitoring point 8 was 0.587 s earlier than that at monitoring point 7. For monitoring point 8, the first overpressure peak appeared with the action of the forward shock wave, whereas the first overpressure peak at monitoring point 7 was caused by the superposition of multiple shock waves. There were strong reactions in the early stage of the explosion at monitoring point 8, while the strong reactions at monitoring point 7 mainly occurred in the middle and late stages.
2. Similarly, the evolution of the explosion shock waves at monitoring point 8 could be divided into four stages. In the first stage, the overpressure did not change very much, and the duration was short. In the second stage, the overpressure continued to increase, and the first overpressure peak of 0.445 MPa was reached at 0.403 s. In the third stage, due to the rising coal dust reaching an explosive concentration, heat gradually accumulated in the pipelines and the reaction was intensified, leading to a significant increase in the peak overpressure. The secondary overpressure of 0.719 MPa was reached at 0.494 s. In the fourth stage, as the attenuation of shock waves outweighed the superposition of shock waves, the overpressure change decreased continuously.
3. Due to the forward shock wave at monitoring point 7, the first overpressure peak of 0.384 MPa was reached at about 0.989 s. Then due to the secondary explosion, the overpressure dropped first and then surged. The second overpressure peak was 0.635 MPa at 1.074 s. At that time, the explosion was in the middle and late stages; that is, the second peak at monitoring point 7 appeared at a later time than that at monitoring point 8. According to the experimental results, we draw the following conclusion: Although monitoring point 7 and monitoring point 8 are both in branch 4, monitoring point 8 is very close to branch 1, monitoring point 7 is far from branch 1, and it is closer to branch 3. At the same time, from the position of the two monitoring points, monitoring point 7 is far from the explosion chamber, and there are more monitoring points that affect the explosion shock wave at monitoring point 7 than monitoring point 8, while monitoring point 8 is more affected by the shock wave at monitoring point 1. Therefore, we get the inference of the analysis in this paper. The reason for determining the peak value of overpressure is based on the distance of monitoring points and the location of each monitoring point in the pipe network.

**Figure 8:**
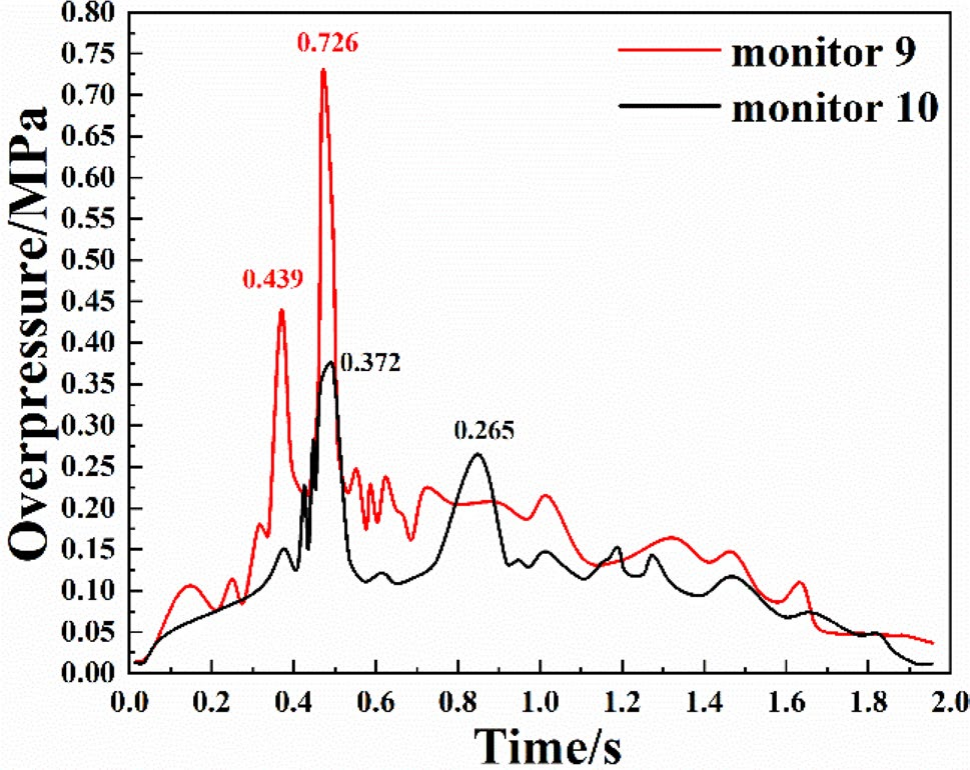
Overpressure change of bifurcation 5.

The variations of the overpressure at two monitoring points in branch pipeline 5 are shown in Fig. 8. It can be seen from the figure that:

1. The shockwave evolution in branch pipeline 5 was similar to that in branch pipeline 2. Both monitoring points reached the first overpressure peak due to the action of the forward shock wave, and the time between the first and second overpressure peaks at both monitoring points was shorter than that in branch pipeline 2. In the evolution process, the fluctuation of the overpressure at monitoring point 9 was larger than that at monitoring point 10. This was because monitoring point 10 was closer to the explosion vent, the overpressure attenuated faster, and the fluctuation range was smaller.
2. The evolution of the shock waves at monitoring point 9 was also divided into four stages. In the first stage, the overpressure fluctuated in a small range and the duration was short. In the second stage, because monitoring point 9 was close to monitoring point 1, the overpressure increased rapidly after a slight fluctuation, and the first overpressure peak was 0.439 MPa at 0.369 s. In the third stage, due to the secondary explosion, the overpressure first dropped and then increased sharply, reaching a peak value of 0.726 MPa at 0.471 s. In the fourth stage, the shock wave was attenuated as the explosion approached its end.
3. The second overpressure peak at monitoring point 10 was smaller than the first peak and the time between the first and second peaks was long. The overpressure rose steadily in the first 0.38 s, yet the increase was not significant. Then the overpressure began to surge at 0.4 s and reached the first overpressure peak of 0.372 MPa at 0.495 s. Next, the overpressure first decreased and then increased, reaching the second peak value of 0.265 MPa at 0.849 s. Because monitoring point 10 was close to the explosion vent and the oxygen content in the pipeline decreased, the second overpressure peak was smaller than the first.

Based on the above analysis, it can be seen that in contrast to straight pipelines or simple bifurcated pipelines^12,15,19,20^, the overpressure did not show a negative correlation with time during the attenuation process^21,22^. Rather, it experienced multiple up-down cycles. Due to the interconnection of pipelines in the pipeline network, the shock wave experienced multiple instances of superposition and offset during the evolution process, resulting in a different overpressure pattern than that in simple pipelines. Thus, the propagation of shock waves in the diagonal pipeline network was a complex process.

**Figure 9:**
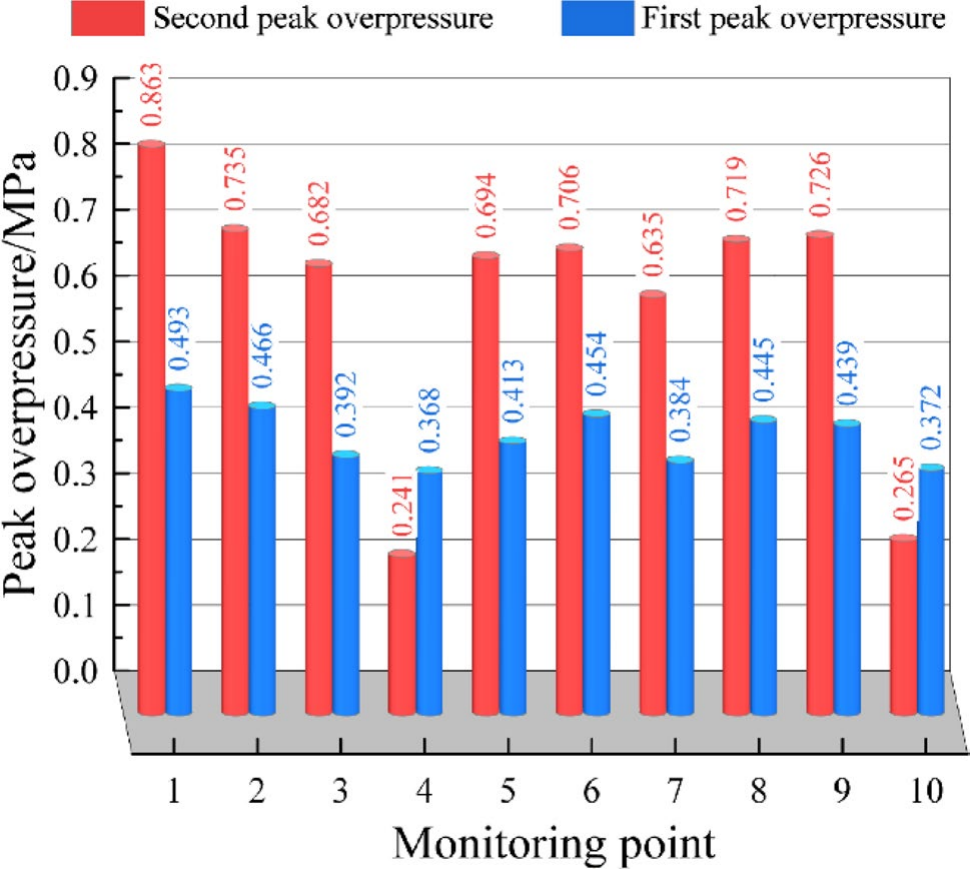
The first and second overpressure peaks at each monitoring point.

Figure 9 shows the first and second overpressure peaks at each monitoring point. It can be seen from the figure that the second overpressure peak at each monitoring point was greater than the first peak, with the exceptions of monitoring point 4 and monitoring point 10. The main reason for this was that both monitoring points 4 and 10 were located close to the explosion vent and far from the explosion source. The consumption of methane, coal dust, and oxygen caused a reduction in the overpressure of the secondary explosion. Thus, the overpressure peak of the secondary explosion was not as high as the first peak. The next section describes how the overpressure attenuation characteristics of the gas–coal dust explosion in the diagonal pipeline network were studied with the overpressure attenuation coefficient and dimensional analysis-based assumptions.

### Overpressure attenuation characteristics

Different overpressure attenuation coefficients were first defined, including the overpressure attenuation coefficient in the straight pipelines *K*_1_*,* the overpressure attenuation coefficient in the branch pipelines *K*_2_, the overpressure attenuation coefficient in the bends *K*_3_, and the overpressure diversion coefficient in the branch pipelines *M*^28^.

**Figure 10:**
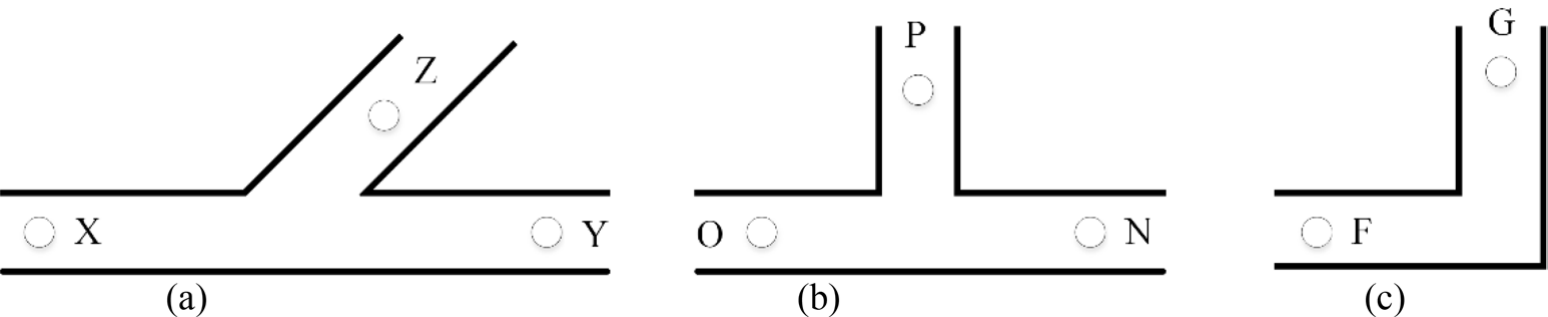
Pipeline types used in the experiment.

The pipeline types included in the experimental network are shown in Fig. 10. In Fig. 10a, K1 = maximum overpressure at monitoring point X/maximum overpressure at monitoring point Y, *K*_2_ = maximum overpressure at monitoring point X/maximum overpressure at monitoring point Z, *M* = maximum overpressure at monitoring point Z/maximum overpressure at monitoring point Y. In Fig. 10b, K1  = maximum overpressure at monitoring point O/maximum overpressure at monitoring point N, *K*_2_ = maximum overpressure at monitoring point O/maximum overpressure at monitoring point P, *M* = maximum overpressure at point P/ maximum overpressure at monitoring point N. In Fig. 10c, K3 = maximum overpressure at monitoring point F/maximum overpressure at monitoring point G.

The overpressure attenuation coefficients are shown in Table 2. It can be seen from the table that some values of *M* and *K*_2_ were less than 1, indicating that due to the influence of the pipeline structure, the overpressure attenuation coefficient in the branch pipeline and the overpressure diversion coefficient in the branch pipeline were different from those in the long, straight pipeline and the simple bifurcated pipeline^29,30^. (The overpressure attenuation coefficient and the overpressure diversion coefficient in the branch pipeline were both greater than 1).

**Table 2:** Overpressure attenuation coefficient at each monitoring point.

Types of overpressure attenuation coefficient	Value
*K*_1_	T_1_/T_2_ = 1.097
	T_6_/T_5_ = 1.017
	T_9_/T_10_ = 2.740
	T_3_/T_4_ = 2.830
	T_8_/T_7_ = 1.132
*K*_2_	T_1_/T_8_ = 1.121
	T_3_/T_5_ = 0.983
	T_9_/T_6_ = 1.028
	T_6_/T_7_ = 1.112
	T_5_/T_7_ = 1.093
*K*_3_	T_2_/T_3_ = 1.078
*M*	T_9_/T_1_ = 0.901
	T_8_/T_2_ = 0.978
	T_5_/T_4_ = 1.886
	T_6_/T_10_ = 1.898
	T_7_/T_5_ = 0.915
	T_7_/T_6_ = 0.899

In terms of *K*_1_, monitoring points 4 and 10 were close to the explosion vent and not connected to the other pipelines, resulting in a low overpressure peak and a larger attenuation coefficient. Without considering monitoring points 4 and 10, the attenuation coefficient from monitoring point 8 to monitoring point 7 was the largest (1.132), indicating that the overpressure attenuation in the diagonal branch pipelines was greater than that in other branch pipelines, and the shock wave is less affected by the pipeline network structure when it is close to the outlet of pipeline network.

For *K*_2_, the attenuation coefficient from monitoring point 3 to monitoring point 5 was less than 1. The increase of the overpressure peak at monitoring point 5 was mainly due to the influence of the pipeline structure. The superposition of shock waves from other pipelines offset the pressure loss from passing through the bifurcated pipeline, and thus, the overpressure attenuation coefficient of the branch pipeline was below 1.

Based on dimensional analysis, it was assumed that the relationship between the overpressure attenuation coefficient $K_{{\text{a}}}$ and the distance *x* between two adjacent monitoring points, the pressure difference $\Delta P$ before and after attenuation, and the pipeline angle variation $\alpha$ were as follows^25,31,32^:

1 $$ K_{a} { = }x^{{\text{A}}} \cdot \Delta P^{{\text{B}}} \cdot \alpha^{{\text{C}}} $$

*A*, *B*, and *C* were nonlinear coefficients to be fitted. Taking the natural logarithm of both sides of Eq. (1), there was:

2 $$ \ln K_{a} {\text{ = A}}\ln x{\text{ + Bln(}}\Delta P) + C\ln \alpha $$

According to Eq. (2), the experimental data were fitted with nonlinear multiple regression, and *A* = 0.0787, *B* =  − 0.0452, and *C* =  − 0.0946.

3 $$ K_{a} { = }x^{0.0787} \cdot \Delta P^{ - 0.0452} \cdot \alpha^{ - 0.0946} $$

The correlation coefficient of Eq. (3) was *R*^*2*^ = 0.906, indicating that it met the accuracy requirements.

In straight pipelines, the overpressure attenuation coefficient is an important parameter to quantify the time-varying characteristics of the explosion overpressure^12,15,19,20^. Similarly, in curved or bifurcated pipelines, the overpressure attenuation characteristics can be optimally quantified using the overpressure attenuation coefficient^14,17,21,22^. After testing, the qualitative conclusions about the overpressure attenuation in some previous studies^12,22,28,30^ also satisfied Eq. (3), indicating that the conclusions obtained in this study could be applied to describe the attenuation of gas–coal dust explosion shock waves in diagonal pipeline networks.

## Conclusions

The propagation and attenuation characteristics of shock waves in a gas–coal dust explosion were studied in a customized diagonal pipeline network. The following conclusions were obtained:

1. The evolution of the overpressure in a gas–coal dust explosion in the diagonal pipeline network was more complicated than those in the straight pipelines and simple branched pipelines. The pressure loss in the diagonal branch pipelines was larger than that in the other branch pipelines.
2. A secondary explosion occurred at each monitoring point. Except for monitoring point 4 and monitoring point 10 that close to the outlet of pipeline network, the second overpressure peak was greater than the first overpressure peak at all other monitoring points. It means the propagation characteristics of shock waves is less affected by the pipeline network structure when it is close to the outlet of pipeline network.
3. Multivariate nonlinear regression was carried out based on the theoretical calculation results, and the relationship between the overpressure attenuation coefficient and the overpressure difference before and after attenuation and the pipeline angle variation was as follows: $K_{{\text{a}}} { = }x^{0.0787} \cdot \Delta P^{ - 0.0452} \cdot \alpha^{ - 0.0946}$.

In this work, we studied the evolution process and characteristics of the gas–coal dust mixed explosion shock wave in the diagonal pipe network. The conclusions obtained from the research can provide reference for the prevention of mine gas explosion accidents and the improvement of the theory of disaster prevention, mitigation and control.

In future research, we plan to study the interaction process between the explosion flame wave and shock wave in detail throughout the explosion process.

## Data Availability

The datasets used during the current study available from the corresponding author (X.T.) on reasonable request.
